# Surface stabilized atorvastatin nanocrystals with improved bioavailability, safety and antihyperlipidemic potential

**DOI:** 10.1038/s41598-019-52645-0

**Published:** 2019-11-06

**Authors:** Manu Sharma, Isha Mehta

**Affiliations:** Department of Pharmacy, Banasthali Vidyapith, Banasthali, 304022 Rajasthan India

**Keywords:** Dyslipidaemias, Drug delivery

## Abstract

Atorvastatin, a favored option for hyperlipidemia exhibits the problem of poor gastric solubility and low absolute bioavailability (12%) along with higher pre-systemic clearance (>80%). Therefore, to circumvent these limitations, atorvastatin nanocrystals were prepared using poloxamer-188 as stabilizer via high pressure homogenization technique followed by lyophilization. Various variables like drug to poloxamer-188 ratio, homogenization cycle, homogenization pressure, type and concentration of cryoprotectant were optimized to achieve uniform nanosized crystals with good dispersibility. Solid state characterization by ATR-FTIR and DSC revealed no incompatible physicochemical interaction between drug and excipients in formulation while DSC and PXRD collectively corroborated the reduced crystallinity of drug in nanocrystals. Size analysis and SEM confirmed nanometric size range of nanocrystals (225.43 ± 24.36 nm). Substantial improvement in gastric solubility (~40 folds) and dissolution rate of drug in nanocrystals was observed. Pharmacokinetic study in wistar rats revealed significant improvement in oral bioavailability (~2.66 folds) with atorvastatin nanocrystals compared to pure drug. Furthermore, reduction in serum total lipid cholesterol, LDL and triglyceride content justified the effectiveness of formulation at 50% less dose of atorvastatin along with improved plasma safety profile in comparison of pure drug. In conclusion, atorvastatin nanocrystals are safe and efficacious drug delivery system confirming potent competence in treatment of hyperlipidemic conditions with ease of scalability for commercialization.

## Introduction

Hyperlipidemia is an idiopathic medical condition arising due to abnormal increase in blood lipids including cholesterol, triglycerides and lipoproteins. It is globally a leading cause of cardiovascular disorders due to formation of atherosclerotic plaque responsible for blockage of uniform blood flow to brain, limbs as well as heart. Several studies have established the correlation between hyperlipidemia and cardiovascular disorders like ischemia, stroke, coronary artery and peripheral vascular disease in today’s era^1^. Atorvastatin is a commonly prescribed statin clinically to reduce elevated level of low density lipoproteins (LDL) cholesterol. It is a competitive inhibitor of HMGCoA reductase involved in biosynthesis of hepatic cholesterol. Inhibition of HMGCoA reductase increases the expression of low density lipoprotein receptors on hepatocytes which facilitates higher LDL uptake and decreases level of LDL cholesterol^2,3^. However, poor solubility, hepatic first pass metabolism, low absolute (12%) and systemic (30%) bioavailability along with higher pre-systemic clearance (>80%) limits its therapeutic efficacy^4^. Food also interferes with rate of absorption (~25%) and extent of absorption (~90%) without affecting LDL-cholesterol lowering efficacy of atorvastatin^5^. Moreover, prolonged use of atorvastatin at high doses enhances susceptibility to severe muscular toxicity like rhabdomyolysis which hinders its wide spread clinical usage^6^.

Commonly utilized strategies for improving solubility and dissolution rate of poorly soluble drugs like atorvastatin involve the use of solubilizers, micronization/nanosizing or transformation of crystalline form to amorphous state^7^. Although, amorphous solids are less stable due to their higher Gibb’s free energy and entropy. Similarly, formulations prepared using higher amount of solubilizers like self emulsifying formulations are not always safe^8^. A variety of colloidal carriers like nanoparticles^9–11^, nanostructured lipid carriers^12^, liposomes^13^, self-emulsifying nanoformulations^14^, reconstituable spray dried ultra-fine dispersion^15^ and lyophilized dry emulsion tablets^16^ have been reported in literature for improving bioavailability and circumscribing the complications associated with atorvastatin pharmacotherapy. However, most of formulations reported are confined either to pharmacokinetic or pharmacodynamic study and deficient in comprehensive details of pharmacokinetic, pharmacodynamic and safety profile. Additionally, poor drug loading capacity, premature drug release, biocompatibility and biodegradability issue of carrier materials as well as difficulty in scalability due to complex manufacturing steps limit their clinical translation.

Recently, nanocrystals have received intense attention as nanotechnology coupled approach to enhance solubility and bioavailability of poorly soluble drugs with extensive commercial eminence due to high drug loading efficiency^17^. Nanocrystallization techniques like milling, precipitation and high pressure homogenization are commonly utilized to prepare nanocrystals. These techniques benefit the most drugs with high crystal lattice energy and solubility less than 200 µg/ml^18^. Among various nanocrystallization techniques, high pressure homogenization technique is a simple, faster, easily scalable and applicable universally for variety of materials^19^. The reduction of particle size in nanometer or submicron range by high pressure homogenization technique dramatically increases effective surface area of particles for interaction with solvent and enhances their saturation solubility and dissolution rate^10,20^. The significant amplification of Gibb’s free energy during nanosizing subsequently facilitates agglomeration of particles to achieve stabilization. Thus, a barrier or stabilizer use becomes essential to ensure sufficient steric or electrostatic repulsion between particles to achieve long term stability of formulation^21^. A stabilizer adsorbed on particle surface stabilizes morphology and size as well as can also amend *in vivo* performance of nanocrystals. Cellulosic polymers (HPC, HPMC, polyvinyl pyrrolidone), poloxamers (PF-68, PF-127), polyethylene glycol, cyclodextrins and surfactants (spans, Tween-80, sodium dodecyl sulfate) have been widely utilized to stabilize colloidal systems alone or in combination as per available literature reports^17,22,23^. Cellulosic polymers being non-toxic and non-irritant can be used to prepare nanocrystals to be delivered by various routes^24^. Their stabilizing efficiency varies according to their molecular weight and viscosity and usually need assistance of surfactants to achieve the desired product^23,25^. However, selection of higher amount of solubilizers like Tween-80, cremophore EL etc may cause hypersensitivity and pain in many patients^26^. Thus, selection of suitable type and amount of stabilizer for development of nanocrystals is most challenging and critical step. Among the stabilizers, poloxamers due to their amphiphilic nature offers better dispersibility and stability to colloidal nanocarriers^21^. Furthermore, poloxamers like poloxamer-188 and 407 have been endorsed as GRAS excipients by USFDA due to biocompatible and non-toxic property to mammalian cells.

Consequently, high pressure homogenization technique to tailor atorvastatin nanocrystals was employed in present study with a goal of improving atorvastatin’s bioavailability and safety. The primary aim of this study was nanonization of atorvastatin, its characterization and evaluation for bioavailability, safety and antihyperlipidemic potential in wistar rats.

## Results and Discussion

### Optimization of atorvastatin nanocrystals formulation

Nanosizing of drug particles often requires high energy input. Therefore, high pressure homogenization technique was utilized to formulate atorvastatin nanocrystals. It was observed that nanosizing of atorvastatin by high pressure homogenization showed significantly smaller average particle size along with uniform particle size distribution and better dispersibility in presence of poloxamer 188 as stabilizer compared to its absence (Table 1). This might be due to physical adsorption of stabilizer over the new surfaces generated during nanosizing which subsequently reduced high surface free energy by stearic stabilization and prevented recoalescence of nanosized particles^23,27^.

**Table 1:** Effect of formulation and process variables on particle size, zeta potential, PDI, drug content and yield of atorvastatin nanocrystals.

Formula-tion Code	Drug: Poloxamer 188 ratio	Homoge-nization pressure	Number of homogen-ization cycles	Particle size (nm ± SD)	Zeta Potential (mV ± SD)	PDI	Drug content (% ± SD)	Yield (% ± SD)
A	Drug only	1000	20	2384.67 ± 69.24	−6.40 ± 3.06	1.00 ± 0.69	99.89 ± 8.34	92.13 ± 6.36
A_1_	1:2	1000	20	914.34 ± 21.32	−12.43 ± 2.56	0.92 ± 0.04	97.01 ± 5.21	89.23 ± 5.64
A_2_	1:3	1000	20	667.25 ± 22.36	−18.63 ± 3.12	0.56 ± 0.05	97.21 ± 4.25	90.23 ± 4.27
A_3_	1:4	1000	20	502.13 ± 25.43	−18.23 ± 2.11	0.41 ± 0.03	98.56 ± 3.21	92.34 ± 3.14
A_4_	1:5	1000	20	225.43 ± 24.36	−24.01 ± 1.96	0.26 ± 0.03	98.97 ± 4.01	94.23 ± 3.56
A_5_	1:6	1000	20	329.56 ± 18.36	−19.36 ± 1.19	0.39 ± 0.02	99.01 ± 3.56	92.21 ± 4.12
A_6_	1:5	1250	20	316.48 ± 34.12	−23.23 ± 2.11	0.32 ± 0.04	98.06 ± 4.17	91.42 ± 4.15
A_7_	1:5	750	20	481.38 ± 27.48	−17.74 ± 3.12	0.42 ± 0.03	99.56 ± 3.86	93.14 ± 2.58
A_8_	1:5	500	20	851.62 ± 32.45	−16.24 ± 2.68	0.87 ± 0.03	99.14 ± 4.24	92.46 ± 3.49
A_9_	1:5	250	20	1366.18 ± 30.48	−15.14 ± 3.04	0.72 ± 0.02	99.42 ± 3.46	93.01 ± 4.27
A_10_	1:5	1000	10	718.23 ± 29.64	−19.41 ± 2.24	0.68 ± 0.03	98.78 ± 3.68	94.23 ± 3.98
A_11_	1:5	1000	30	284.56 ± 30.14	−21.35 ± 2.45	0.46 ± 0.02	99.14 ± 3.56	92.12 ± 4.18

Subsequently, the concentration of poloxamer 188 was further optimized to achieve desirable particle size with good dispersibility. The average particle size and polydispersity index ranged 225.43 ± 24.36 nm to 914.34 ± 21.32 nm and 0.26 ± 0.03 to 0.92 ± 0.04 respectively on altering drug: poloxamer ratio from 1:5 to 1:2. It was observed that increase in concentration of poloxamer 188 facilitated smaller size particles (Table 1). The outcomes of study established that adsorption of hydrophobic domains of poloxamer 188 and micellar structure formed by hydrophilic domains (poly-ethylene oxide chains) impeded aggregation and crystal growth^28^. Higher average particle size and polydispersity index of batches prepared with lower concentration of stabilizer confirmed the insufficiency of poloxamer 188 to reduce the surface free energy of new surfaces generated and their growth into bigger crystals. However, further increase in stabilizer concentration relative to drug had led to genesis of larger size particles due to increased viscosity of system counteracting the shear forces experienced by formulation during homogenization (Table 1)^26^.

Process parameters like hydraulic homogenization pressure and number of cycles had also showed significant effect on particle size and polydispersity index (Table 1). Homogenization pressure disrupted large particles and their aggregates simultaneously due to induced phenomenon of cavitation, shear and turbulence in Microfluidizer (M-110P; Microfluidics, MA, USA). Thus, provides large surface area for stabilizer to be adsorbed and form micellar structures. Significant nanosizing of drug was observed on increasing the homogenization pressure from 250 bars to 1000 bars. However, no remarkable effect on particle size diminution was observed beyond 1000 bars. This might be attributed to high energy input at higher homogenization pressure contributing to re-aggregation of particles causing stagnancy in downward trend of particle size reduction^29^. Similarly, increase in homogenization cycles from 10 to 20 cycles had reduced the average particle size of nanocrystals significantly along with their PDI. Increasing number of homogenization cycles increases the probability of particles to pass through the zone of higher power density in Microfluidizer and thus greater diminution of particle size. However, further increase in homogenization cycle i.e. above 20 cycles had no marked effect on particle size and PDI. Homogenization cycles also had not showed any considerable effect on zeta potential of nanocrystals. During homogenization at higher homogenization pressure (>1000 bars) and large number of cycles (>20 cycles), recoalescence rate increased. Higher particles collision rate compared to physical adsorption rate of stabilizer over the newer surfaces generated might have contributed to enhanced recoalescence. Thus, increased energy input had increased the particle size rather than the expected smaller sizes^30,31^. Good yield (89.23 ± 5.64% to 94.23 ± 3.98%) and atorvastatin content (97.01 ± 5.21% to 99.56 ± 3.86%) was achieved for all batches. However, inconsiderable reduction in both yield and drug content might be due to processing losses occurring during atorvastatin nanocrystal production. Drug content of all batches prepared was within ± 5% of theoretical amount which indicated suitability and reproducibility of atorvastatin nanocrystal preparation method. Finally, optimized formulation was prepared by homogenizing drug to poloxamer ratio 1:5 for 20 cycles at 1000 bars for further studies.

### Cryoprotectant selection

Lyophilization of aqueous dispersion of nanocrystals was performed to transform them into stable and easily re-dispersible dry state. Therefore, various cryoprotectants at different concentrations were screened to prevent crystal growth and facilitate ease of re-dispersibility of nanocrystals upon reconstitution with water. It was observed that both type as well as concentration of cryoprotectant affected the particle size, zeta potential and PDI of re-dispersed formulation (Table 2). Moreover, higher particle size and PDI was perceived for formulations lyophilized at lower concentration of cryoprotectant i.e. 5% w/v of mannitol or trehalose or lactose on re-dispersion indicating aggregation and crystal growth during process of freeze drying. Formulations lyophilized with 10% w/v mannitol showed ease of redispersion without agglomerates. The ability of cryoprotectant to protect formulation from aggregation followed the order mannitol > trehalose > lactose (Table 2). Slight increase in particle size was observed during freeze drying even with optimal mannitol concentration (10% w/v) although increase in particle size and PDI was not significant compared to freshly prepared formulations. Such increase in particle size during lyophilization might be contributed by thermodynamic tendency of particles to aggregate during freeze drying even though osmotic activity of mannitol counteracts to a great extent. The results of present study are consistent with earlier published results^32^. Eventually, the optimized formulation lyophilized using mannitol (10% w/v) as cryoprotectant was selected for further studies.

**Table 2:** Physicochemical properties of re-dispersed atorvastatin nanocrystals containing different cryoprotectants.

Type of cryoprotectant	Amount (%)	Appearance	Particle size (nm ± SD)	Zeta Potential (mV ± SD)	PDI	Re-dispersion time (sec)
None	0	Collapsed	1356.75 ± 93.67	−11.2 ± 3.72	> 1	>120
Mannitol	5	Slightly Collapsed	384.65 ± 18.23	−24.31 ± 2.65	0.45 ± 0.01	45
	10	Uniform	258.43 ± 15.45	−26.64 ± 3.17	0.23 ± 0.02	29
Trehalose	5	Slightly collapsed	437.31 ± 18.65	−21.31 ± 4.67	0.34 ± 0.01	60
	10	Uniform	326.14 ± 16.08	−24.14 ± 3.61	0.26 ± 0.03	42
Lactose	5	Collapsed	628.32 ± 14.23	−16.45 ± 7.63	0.68 ± 0.03	118
	10	Slightly collapsed	394.61 ± 15.41	−21.23 ± 16.51	0.38 ± 0.02	93

### ATR-FTIR

**Figure 1:**
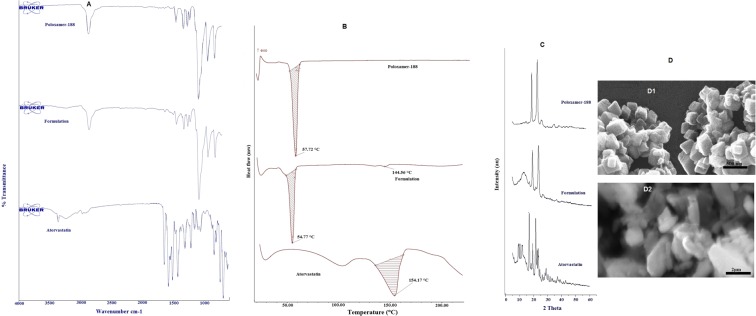
**(A)** ATR-FTIR spectra of atorvastatin, poloxamer 188 and optimized atorvastatin nanocrystals respectively. **(B)** DSC thermograms of atorvastatin, poloxamer 188 and optimized atorvastatin nanocrystals respectively. **(C)** Powder X- ray diffractograms of atorvastatin, poloxamer 188 and optimized atorvastatin nanocrystals respectively. **(D)** Scanning electron micrographs of optimized nanocrystals (D1) and pure drug (D2).

ATR-FTIR spectrum of pure atorvastatin exhibited distinctive peaks at 3362.72 cm^−1^ due to N-H stretching, 3241.51 cm^−1^ due to symmetric O-H stretching, 2971.04 cm^−1^ due to CH-stretching, 1649.52 cm^−1^ due to asymmetric C=O stretching, 1580.85 cm^−1^ and 1515.31 cm^−1^ result from N-H bending and C-N stretching of C-N-H group, 1430.01 cm^−1^ and 1376.76 cm^−1^ due to O-H bending and C-O stretching of carboxylic acid and 1217.51 cm^−1^ due to aromatic C-N stretching. Poloxamer 188 showed an intense overlapped band at 1097.66 cm^−1^ due to asymmetric C-O-C stretching. Spectrum of atorvastatin nanocrystals exhibited characteristic peak of atorvastatin at 3232.87 cm^−1^, 1646.77 cm^−1^ and 1513.59 cm^−1^ due to symmetric O-H stretching, asymmetric C=O stretching and C-N-H group respectively as well as poloxamer 188 at 1097.92 cm^−1^ due to asymmetric C-O-C stretching. Comparison of spectrum of atorvastatin with atorvastatin nanocrystals exhibited no remarkable difference in position of peaks for atorvastatin confirming the compatibility of drug with excipients in formulation. However, concomitant decline in the intensity of respective peaks designated for atorvastatin was observed in spectrum of formulation due to dilution effect of higher concentration of poloxamer 188 in formulation (Fig. 1A).

### Thermal analysis

Thermal studies were performed to evaluate additional properties of drug alone and in naocrystal formulation (Fig. 1B). Atorvastain showed a characteristic sharp endothermic peak at 154.17 °C whereas poloxamer 188 exhibited broad endothermic peak at 57.72 °C. Poloxamer 188 based atorvastatin nanocrystals exhibited characteristic peaks at 54.77 °C and 144.56 °C which respectively symbolized the presence of poloxamer 188 and drug in formulation. The decrease in melting point might be contributed by reduction in crystal size and crystal lattice energy of atorvastatin in nanocrystals due to incorporation of drug in hydrophobic domain of micelles formed by stabilizer^33,34^.

### PXRD analysis

Diffractograms of atorvastatin, poloxamer 188 and optimized formulation are presented in Fig. 1C. The unprocessed atorvastatin showed characteristic intense and sharp diffraction peaks at 2θ values between 11 and 32° indicating its crystalline behavior. The presence of characteristic peaks of atorvastatin in diffractogram of optimized formulation represented that high-pressure homogenization did not disturb the drug crystallinity. However, peak broadening along with reduction in peak intensity indicating reduced crystallinity of drug was observed due to complete surface coverage of drug particles by poloxamer 188^35,36^. The results of thermal and diffraction studies ensured the reduced crystallinity of drug in nanocrystals which might have attributed to its enhanced solubility and dissolution.

### Morphology

SEM images of pure drug divulged irregular crystalline shape of particles in the size range of 2 to 3 µm. While optimized formulation exhibited homogeneous cubical shaped nanocrystals in a size range of 170–240 nm (Fig. 1D). Imaging results are in good agreement with mean particle size and PDI determined by NanoZS Malvern indicating that all formulation and process variables had been suitably optimized. It also indicated that inclusion of poloxamer 188 only stabilized the crystals in their nanosized physical state without altering the crystallinity of drug^37^.

### Solubility studies

The aqueous solubility of atorvastatin and optimized lyophilized formulation in water, HCl buffer pH 1.2 (simulated gastric pH) and phosphate buffer pH 6.8 (simulated intestinal pH) are shown in Table 3. Improved saturation solubility of drug by nanosizing using poloxamer-188 as stabilizer was observed at pH 1.2 (~40 folds), pH 6.8 (~20 folds) and water (~18 folds). This tremendous rise in solubility of atorvastatin showed excellent affinity between atorvastatin and poloxamer 188 to form molecular dispersion responsible for changing solubility equilibrium and saturation solubility of drug. Furthermore, improvement in drug solubility was contributed by nanosizing of atorvastatin in presence of stabilizer which provided stable atorvastatin nanocrystals with higher surface area for interaction with aqueous phase facilitating improved wetting and dispersibility^38^.

**Table 3:** Saturation solubility of drug and optimized atorvastatin nanocrystals in different media.

Sample	Drug: Poloxamer 188 ratio	Amount of drug solubilized (µg/ml)		
		HCl Buffer pH 1.2	Water	Phosphate buffer pH 6.8
Atorvastatin drug	—	1.27 ± 6.17	12.67 ± 3.24	13.01 ± 4.79
Atorvastatin nanocrystals	1:5	50.14 ± 5.46	226.45 ± 7.35	248.67 ± 8.12

### Dissolution studies

**Figure 2:**
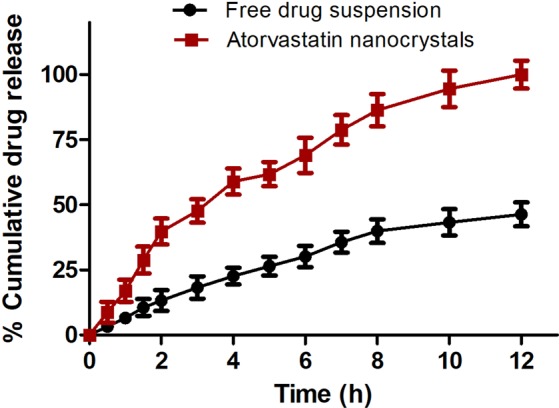
*In vitro* release behavior of drug and atorvastatin nanocrystals in pH progressive media respectively.

*In vitro* release study bestows practical comprehension into expected *in vivo* behavior of developed dosage form. Atorvastatin nanocrystals showed burst cumulative drug (~40%) release in 2 h followed by prolonged release upto 12 h (Fig. 2). This elevated rate of atorvastatin release might be contributed by stable nanosize of optimized formulation leading to poor agglomeration of nanocrystals, good wetting and dispersibility. The presence of stabilizer at interface of drug and aqueous phase also reduced surface tension between them by interaction of ether oxygen of polyethylene oxide blocks of poloxamer 188 via hydrogen bonding with water molecules^21^. However, prolonged release might be attributed to formation of multimolecular micelles of poloxamer 188. Hydrophobic domain of micelles might have interacted with atorvastatin via Van der Waals forces and slowed down partitioning and diffusion of drug from the core multimolecular micelles^39^.

### Stability studies

Optimized formulation stored at room and accelerated temperature respectively for 6 months was assessed for physical and chemical stability to identify the stability boundaries in support of its storage recommendation. The results of stability study of samples stored at room temperature showed no remarkable change in particle size, PDI and atorvastatin content. However, considerable alteration in particle size and PDI was detected following 6 months of storage at accelerated conditions (Table 4). The crystal growth on storage at 40 °C might be contributed by Ostwald ripening^40^. Although drug content remained above 95% at both storage conditions indicating that lyophilized atorvastatin nanocrystals were stable with no drug degradation. Furthermore, results confirmed that use of high pressure homogenization technique for nanosizing of atorvastatin had not affected the chemical stability of atorvastatin.

**Table 4:** Stability studies of optimized atorvastatin nanocrystals under room temperature (25 ± 2 °C/60 ± 5% RH) and accelerated (40 ± 2 °C/75 ± 5% RH) storage conditions.

S. No.	Storage condition	Storage time (month)	Particle size (nm ± SD)	Zeta Potential (mV ± SD)	PDI
1.		0	258.43 ± 15.45	−26.64 ± 3.17	0.23 ± 0.02
2.	Room temperature (25 ± 2 °C/60 ± 5% RH)	1.5	263.14 ± 15.21	−25.01 ± 3.52	0.24 ± 0.02
		3	281.75 ± 16.34	−23.20 ± 4.32	0.21 ± 0.02
		6	304.21 ± 17.14	−22.94 ± 5.67	0.26 ± 0.03
3.	Accelerated temperature (40 ± 2 °C/75 ± 5% RH)	1.5	289.67 ± 12.26	−23.67 ± 3.52	0.25 ± 0.04
		3	328.74 ± 17.69	−22.04 ± 4.56	0.29 ± 0.03
		6	406.38 ± 14.96	−17.01 ± 4.83	0.51 ± 0.02

### Pharmacokinetics

**Figure 3:**
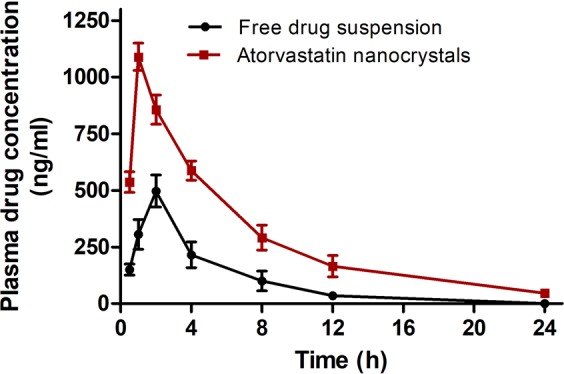
Plasma drug concentration versus time profiles after oral administration of atorvastatin dispersion and atorvastatin nanocrystals respectively.

Plasma concentration time profile of atorvastatin suspension and optimized formulation after single oral dose equivalent to 20 mg/Kg atorvastatin have been co-plotted in Fig. 3 while pharmacokinetic parameters are presented in Table 5 respectively. Relatively higher C_max_ (2.19-fold) and AUC_0–24_ (2.66-fold) of atorvastatin nanocrystals confirmed higher rate and extent of absorption compared to atorvastatin suspension. Improved solubility and dissolution rate of atorvastatin offered by optimized formulation might have contributed high drug concentration gradient between GIT and blood vessels which accounted for higher atorvastatin plasma concentration^22^. Furthermore, poloxamers potential of permeability enhancement by altering micro-viscosity of cellular membrane also contributed to higher bioavailability^41^. Immediate absorption depicted by significantly lower T_max_ and higher plasma concentration at all time points in 24 h profile of atorvastatin nanocrystals was observed compared to atorvastatin suspension. Oral administration of atorvastatin nanocrystals extended elimination half-life and MRT (1.42-fold) of atorvastatin compared to atorvastatin suspension indicated slower systemic clearance. This might be contributed by substantial lymphatic absorption of nanocrystals via paracellular pathways and phagocytosis of colloidal sized particles across the GIT where atorvastatin nanocrystals might dissolve slowly and diffuse down the concentration gradient to reappear in blood over several hours similar to classical redistribution^42^.

**Table 5:** Plasma pharmacokinetic parameters after oral administration of atorvastatin dispersion and atorvastatin nanocrystals in wistar rats (20 mg/Kg atorvastatin).

Parameters	Atorvastatin	Atorvastatin nanocrystals
T_max_ (h)**	2.00 ± 0.21	1.00 ± 0.16
C_max_ (ng/ml)**	497.01 ± 71.23	1089.78 ± 59.89
t_1/2_ (h)**	2.73 ± 0.06	5.07 ± 0.08
k_e_ (h^−1^)**	0.25 ± 0.03	0.14 ± 0.01
AUC_(0–24)_ (ng h/ml)**	2446.30 ± 80.21	6518.15 ± 101.16
AUMC (ng h^2^/ml)**	5559.46 ± 84.13	21034.60 ± 93.13
MRT (h)*	2.27 ± 0.27	3.23 ± 0.35
Relative bioavailability (%)	—	266.45

*p < 0.05 level of significant difference; **p < 0.001 level of significant difference.

Various strategic approaches for enhancement of atorvastatin bioavailability, a prerequisite for the success of atorvastatin formulation are available in literature. However, some of them has narrow accomplishment to enhance the absorbed fraction of the atorvastatin dose like spray drying (~1.98 fold)^11^, self-emulsifying system (~1.93 fold)^14^, nanosuspension (~1.55 fold)^14^, gastro-resident formulation (~1.68 fold)^43^, solid self-emulsifying system (~2.16)^44^, submicron emulsion (~2.58 fold)^45^, solid dispersion with polyvinylpyrrolidone vinyl acetate (~1.38 fold)^46^ or hydroxypropyl methyl cellulose (~1.68 fold)^47^. In present study, atorvastatin processed with highly hydrophilic polymer i.e. poloxamer 188 augmented bioavailability enhancement consistent to relative boost of dissolution of atorvastatin nanocrystals. The outcomes of pharmacokinetic study of atorvastatin nanocrystals disclosed promising prospective of nanosizing in atorvastatin delivery with a sense of control over atorvastatin plasma concentration for prolonged period as disclosed by 2.66-fold higher relative bioavailability compared to pure drug.

### *In vivo* efficacy

In the present study, high fat diet was used to induce hyperlipidemia. Since prolonged consumption of high fat may create diverse pattern of hyperlipidemia due to increased synthesis of triglycerides along with inhibition of β-oxidation of fatty acids which consequently leads to accumulation of excess triglycerides in liver. Such idiosyncrasies in lipid metabolism are typically corelated with cardiac disorders, obesity and related diseases^48,49^.

During high fat diet containing egg yolk and lard as source of saturated fatty acid, weight gain, serum total cholesterol and LDL level was significantly high in control group. However, remarkable reduction in total cholesterol, LDL, VLDL and triglyceride (TG) level was observed on oral treatment with drug and optimized formulation at p < 0.05 compared to control group (Table 6). This was contributed due to inhibition of expression of HMG-CoA reductase involved in catalysis of a rate limiting step in cholesterol synthesis. The effect of optimized formulation on lowering serum lipid level was significantly equal to crude drug at 50% less dose of atorvastatin. This might be attributed by the nanosize and presence of poloxamer 188 on the surface of nanocrystals facilitating enhanced endocytic uptake^42^. Furthermore, the inhibitory effect of poloxamer 188 on P-glycoproteins and CYP3A4 enzymes as well as capability of changing microviscosity of cellular membrane might have improved cellular uptake of atorvastatin^21,50^. Thus, enhanced *in vivo* efficacy of optimized formulation might be attributed by improved uptake, prolonged circulation of nanocrystal in blood and sustained release behavior of formulation maintaining therapeutic drug concentration systemically with prolonged inhibitory effect on HMG-CoA reductase involved in lipid synthesis.

**Table 6:** Plasma lipid and safety profile of animals treated with crude drug and optimized formulation.

S. No.	Biochemical variables	Control	HFD	Drug	Formulation
1.	Total Cholesterol (mg/dl)	106.56 ± 17.91	240.89 ± 21.95^***a^	181.85 ± 12.28^**a,*b^	162.65 ± 11.68^*a,**b^
2.	LDL (mg/dl)	55.31 ± 9.94	146.97 ± 11.13^***a^	106.77 ± 10.84^**a,**b^	88.95 ± 7.41^*a,**b^
3.	HDL (mg/dl)	27.38 ± 3.43	9.25 ± 1.54^***a^	19.21 ± 1.99^*a,**b^	24.21 ± 3.01^***b^
4.	Triglyceride (mg/dl)	85.10 ± 4.02	196.42 ± 11.27^***a^	95.34 ± 2.98^***b^	88.55 ± 2.45^***b^
5.	VLDL (mg/dl)	17.02 ± 0.80	39.28 ± 2.35^***a^	19.07 ± 0.60^***b^	17.71 ± 0.49^***b^
6.	Creatinine kinase (U/l)	113.53 ± 15.15	116.86 ± 11.09	128.31 ± 8.71	123.71 ± 5.11
7.	Plasma creatinine (mg/dl)	1.14 ± 0.19	1.52 ± 0.12	3.87 ± 0.53^***a,***b^	2.52 ± 0.36^**a,*b,**c^
8.	LDH (U/l)	254.72 ± 22.67	261.36 ± 21.28	383.86 ± 35.22^**a,**b^	304.56 ± 18.30^*c^
9.	Urea (mg/dl)	10.23 ± 0.83	11.06 ± 1.35	18.43 ± 3.16^**a,*b^	12.54 ± 1.61^*c^

^***^P < 0.001, ^**^P < 0.01, ^*^P < 0.05, ^a^versus control, ^b^versus HFD (high fat diet), ^c^versus Drug.

### Safety evaluation

Prolonged clinical use of atorvastatin often imparts skeleton muscle myopathy, myalgia progressing to rhabdomyolysis due to its accumulation in non-hepatic tissues. Therefore, plasma level of various biomarkers like creatinine kinase (CK), lactate dehydrogenase (LDH), creatinine and urea were estimated to diagnose skeleton muscle toxicities in rats treated with drug and formulation for 2 weeks. No significant change in creatinine kinase level was obtained in both drug and formulation treated groups. Although significant elevation of LDH, creatinine and urea plasma level were observed in drug treated group compared to formulation (Table 6). Improved safety profile of atorvastatin nanocrystals might be accredited to lower atorvastatin dose and their sustained release behavior which also protected drug from rapid metabolism. Thus, ameliorated lipid lowering potential of atorvastatin nanocrystals at half of dose documented in literature for various reported formulations like re-constituable spray dried ultra-fine atorvastatin dispersion^15^, lyophilized dry emulsion tablets^16^ and atorvastatin solid dispersion^51^ with good safety index confirmed the suitability and novelty of formulation in regulating hyperlipidemia.

**Figure 4:**
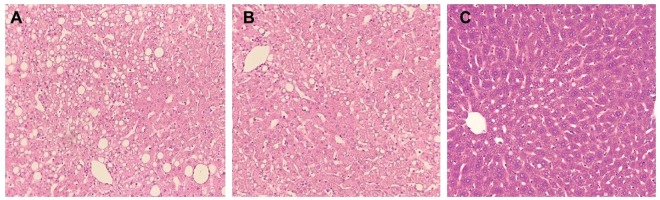
Histopathological changes in liver of high fat diet control group (**A**), Drug treated (**B**) and optimized formulation treated group (**C**).

Histopathological evaluation of liver of formulation treated animals also revealed lower accumulation of lipid droplets in liver tissues compared to drug treated and control animals. Numerous fat droplets and severe vascular changes in liver tissues of control group animals were observed (Fig. 4). No significant change in liver cells was observed in all treated groups confirming no harmful effect of drug and formulation respectively.

## Materials and Methods

### Materials

Atorvastatin and poloxamer 188 were obtained as gratis sample from Merck (Mumbai, India) and Jubilant Life Sciences Ltd. (Noida, India) respectively. Lactose, mannitol, trehalose, potassium dihydrogen ortho phosphate and disodium hydrogen ortho phosphate were purchased from S.D Fine chemicals Ltd. (Mumbai, India). HPLC grade acetonitrile and water was purchased from Merck Life Science Pvt. Ltd. Double distilled water was used all over the study.

### Preparation of atorvastatin nanocrystals

Briefly, atorvastatin was dispersed in aqueous solution of poloxamer 188 using ultra turrax (IKAT 25, Germany) for 1 min. Subsequently, atorvastatin dispersion was processed using high shear fluid processor equipped with heat exchanger (Microfluidizer M-110P; Microfluidics, MA, USA) for 20 cycles at 1000 bars. The resultant suspension was lyophilized (Labconco benchtop freeze dryer system, MO, USA) after addition of suitable amount of cryoprotectant. Lyophilized atorvastatin nanocrystals were stored at 4 °C till further use. Several batches of atorvastatin nanocrystals were prepared by varying various formulation (concentration of stabilizer, type and concentration of cryoprotectant) and processing variables (homogenization pressure and number of cycles) as shown in Table 1 to confirm the optimum conditions for atorvastatin nanocrystals formation.

### Physicochemical characterization of nanocrystals

#### Size and zeta potential

The average particle size diameter and zeta potential of different batches of atorvastatin nanocrystals along with their distribution was measured utilizing Malvern Zeta Sizer (Nano ZS, Malvern Instruments, UK). Samples were appropriately diluted and dispersed in distill water for 1 min using vortexer before analysis.

### Drug content and yield determination

Lyophilized atorvastatin nanocrystals (20 mg) were dissolved in methanol and passed through syringe filter (0.22 µm pore size). The collected filtrate was diluted adequately before analyzing spectrophotometrically at 247 nm.

The percent yield of a batch was determined by dividing the weight of lyophilized atorvastatin nanocrystals obtained by total weight of all solids used to prepare lyophilized nanocrystals.

### Cryoprotectant selection

Traditional cryoprotectant like mannitol, trehalose and lactose with different concentration i.e. 5% w/v and 10% w/v were respectively employed to evaluate the suitable cryoprotectant for the formulation. Different batches prepared after dissolving suitable cryoprotectant into liquid aqueous atorvastatin nanocrystal formulation respectively were frozen at −60 °C for 12 h. Afterwards, frozen samples were lyophilized at 0.07 mbar for 24 h using lyophilizer (Labconco benchtop freeze dryer system, MO, USA).

Re-dispersibility of freeze-dried samples was evaluated by adding milli Q water (10 ml) to lyophilized cake with manual shaking for 1 min. Subsequently, samples were sonicated in bath sonicator for 1 min and analyzed for particle size, PDI and zeta potential.

### Solubility determination

Aqueous solubility of drug and optimized lyophilized batch of atorvastatin nanocrystals was evaluated using conventional shake flask method. Briefly, an excess amount of sample added to deionized water, HCl buffer pH 1.2 and phosphate buffer pH 6.8 respectively were shaken at 37 ± 1 °C for 24 h. Aliquots (2 ml) were withdrawn and centrifuged at 15,000 rpm for 20 min. Supernatants collected were syringe filtered (0.22 µm pore size) and analyzed spectrophotometrically.

### Morphology

Morphology of pure drug and lyophilized atorvastatin nanocrystals was determined by field emission scanning electron microscopy (MIRA 3 TESCAN). Samples were clanged on a double adhesive tape and sputter coated with gold and palladium under an inert environment of argon. Samples were scanned and visualized through field emission scanning electron microscope at an excitation voltage of 5 KV and photomicrographs were captured.

### Solid state characterization

ATR-FTIR spectroscopy was used to determine the possible chemical interaction between drug and excipients used in preparation of atorvastatin nanocrystals. ATR-FTIR spectra were recorded with in a spectral region of 4000–400 cm^−1^ at a resolution of 4 cm^−1^ with a scanning frequency of 45 times.

Thermal behavior of drug, poloxamer 188 and optimized formulation was analyzed utilizing differential scanning calorimeter DSC-60 (Shimadzu, Japan). An accurately weighed amount of samples (2–3 mg) were crimped into aluminum pans and heated over a range of 40–250 °C at a scanning rate of 10 °C min^−1^ under a continuous nitrogen purge (40 ml/min).

X-ray diffraction study was performed to evaluate crystallinity of atorvastatin, poloxamer 188 and optimized formulation utilizing Philips PAN analytical expert PRO X-ray diffractometer 1780 (Netherlands). Instrument was operated at 40 kv, 40 mA with Cu-Kα line as radiation source. All samples were scanned over a 2Ө range between 5° to 60° with a scan rate of 5°/min.

### *In-vitro* dissolution studies

Dissolution behavior of atorvastatin and atorvastatin nanocrystals was analyzed in pH progressive dissolution media (HCl buffer pH 1.2 for 2 h followed by phosphate buffer pH 6.8) utilizing paddle type USP dissolution apparatus. An accurately weighed amount of drug/drug nanocrystals corresponding to 50 mg drug was kept in dialysis bag (12 KDa Molecular weight cut off, Himedia, India). Subsequently, dialysis bags were suspended in release media (900 ml) containing 0.1% w/v Tween-80 to simulate sink conditions under continuous stirring (75 rpm) in thermostatically controlled condition (37 ± 1 °C). Aliquots (3 ml) were drawn out at predestined time intervals upto 12 h with subsequent renewal with fresh dissolution media to keep the dissolution fluid volume constant. The collected samples were syringe filtered (0.22 µm) and analyzed spectrophotometrically at 247 nm.

### Stability study

Optimized formulation was monitored for stability according to ICH guidelines. Samples were imposed to different storage conditions i.e. 25 ± 2 °C at 60 ± 5% RH and 40 ± 2 °C at 75 ± 5% RH for 6 months. All the samples were evaluated for change in particle size, zeta potential and polydispersity index after re-dispersing lyophilized formulation at an interval of 1.5, 3 and 6 months along with visual observation for physical stability.

### *In vivo* study

Three-month old wistar rats (150–200 g) were received from the animal house of Department of Pharmacy, Banasthali Vidyapith, Rajasthan, India. All *in vivo* studies were approved by the Institutional Animal Ethical Committee of Banasthali Vidyapith, Rajasthan (574/GO/ReBi/S/02/CPCSEA) and carried out in accordance with CPCSEA guidelines and regulations, Ministry of Social Justice and Empowerment, Government of India. Animals housed in polypropylene cages were acclimatized on normal animal feed (commercial pellets) and water *ad libitum* at 22 ± 1 °C and 45–55% RH with photoperiodic exposure of light and dark cycle of 12 h each respectively.

### Pharmacokinetic study

Twelve wistar rats were randomly distributed into two groups (n = 6). Atorvastatin (20 mg/kg) and atorvastatin nanocrystals (equivalent to 20 mg/kg atorvastatin) dispersed in 0.3% w/v sodium carboxy methyl cellulose was given orally to overnight-fasted rats of group I and group II respectively. Blood samples (250 µl) were collected from rat tail vein in heparinized tube at 0.5, 1, 2, 4, 8, 12 and 24 h post-dosing. Subsequently plasma was separated from blood samples by centrifuging them at 5000 rpm for 10 min at 4 °C. Separated plasma was stored at −60 °C until quantified by RP-HPLC (LC-2010CHT; Shimadzu, Japan). Mobile phase composed of acetonitrile and phosphate buffer pH 5.0 (45:55 v/v) at 25 ± 2 °C with an adjusted flow rate of 1.0 ml/min was used to achieve HPLC resolution of samples. The eluted atorvastatin was analyzed at 247 nm^9^.

The data of plasma drug concentration with respect to time was evaluated utilizing WinNonlin software (5.1: Pharsight, Mountain View, CA).

### *In vivo* efficacy and safety

High fat diet composed of commercial rat chow (75.5%), egg yolk (12.5%), lard (8.5%), cholesterol (3%) and bile salt (0.5%) respectively were mixed, pelleted and provided to wistar rats for 4 weeks to instigate hyperlipidemia. Blood samples collected from caudal vein of animals maintained on high fat diet were evaluated for serum lipid level^52^. A considerable elevation in serum lipid level indicated the establishment of hyperlipidemia with high fat diet. Twelve animals were randomly segregated into three groups (n = 4): group I: positive control (orally treated with 0.5% carboxymethyl cellulose sodium solution); group II: atorvastatin suspension treated (oral, 5 mg/Kg); group III: optimized formulation treated (oral, equivalent to 2.5 mg/Kg atorvastatin). Animals of each group were treated with respective regimen consecutively for 2 weeks after induction of hyperlipidemia. Blood samples collected after 2 weeks treatment from respective group were centrifuged at 5000 rpm for 10 min to separate plasma. The collected plasma samples were analyzed for various biochemical parameters like total cholesterol (TC), low density lipoprotein (LDL), high density lipoprotein (HDL), triglyceride level, plasma creatinine kinase, creatinine and urea level using commercially available diagnostic kit (Span Diagnostic Ltd., Surat, India).

Animals were sacrificed on day 14 of treatment and liver was excised, weighed and analyzed for histological lesions. Liver tissues were fixed in 10% formalin at room temperature. Tissues embedded in paraffin were sectioned (3–4 mm thick), mounted on glass slides and stained using haematoxylin and eosin dye and observed for histological changes under light microscope fitted with image analyzer.

### Statistical analysis

Data are demonstrated as mean ± SD. Statistical significance of differences between means was analyzed by one-way ANOVA followed by Bonferroni test for comparisons of related data using GraphPad Prism Software (version 5.03, GraphPad Software Inc CA, USA) at p < 0.05.

## Conclusions

The development of atorvastatin nanocrystals was successfully achieved. Optimized atorvastatin nanocrystals exhibited remarkable enhancement in solubility and bioavailability with prolonged biological half life. Serum lipid profiles had demonstrated higher potential of atorvastatin nanocrystals to lower hyperlipidemia even at 50% lesser dose compared to drug suspension. Furthermore, formulation restored more meaningfully liver tissue histology after intoxication with cholesterol rich diet. Thus, preparation of nanocrystals of poorly soluble drug like atorvastatin is a consistent and effective approach for improving its oral bioavailability, safety and bioactivity with ease of commercial scalability.
